# A causality investigation into stock prices and macroeconomic indicators in the Indian stock market

**DOI:** 10.12688/f1000research.157041.1

**Published:** 2024-10-30

**Authors:** Sanjay Singh Chauhan, Pradeep Suri, Debapriyo Nag, Farman Ali

**Affiliations:** 1 Management, Uttaranchal University, Dehradun, Uttarakhand, 248007, India; 2 Management college of southern Africa, Honoris, United Universities, Durban, Durban, Durban, South Africa

**Keywords:** Indian Stock markets, Macroeconomic variables, Causal relationships, Stock prices, ARDL

## Abstract

**Background of the study:**

The systematic impact of macroeconomic variables on stock market returns makes it crucial to comprehend the link between macroeconomic variables and the stock market. Stock prices are closely linked to macroeconomic indicators, a crucial aspect for investors, policymakers, and researchers in emerging markets like India, influencing investment decisions and policy formulation.

**Methods:**

The autoregressive distributed lag (ARDL) model was used in this study to examine the causal links between specific macroeconomic factors and Indian stock prices from April 2009 to March 2023.

**Results:**

The outcomes of the research suggest that macroeconomic variables exert influence on the Indian stock market, across the short and long term. Moreover, the results of the paired Granger causality test suggest that the domestic macroeconomic variables possess predictive significance for stock prices in the Indian stock market.

**Conclusion:**

The study reveals that macroeconomic variables significantly impact the Indian stock market, highlighting the need for investors and portfolio managers to monitor these conditions to optimize returns and mitigate risks. The Reserve Bank of India should maintain an optimal money supply to prevent inflation and exchange rate fluctuations, while bolstering the export sector and facilitating imports through initiatives like Atma-nirbhar Bharat Abhiyan and Make in India. Policies focusing on productivity, infrastructure, and a favourable business environment are also crucial. Therefore, it is crucial for investors and portfolio managers to consistently analyse the current macroeconomic conditions in order to maximize their profits and minimize risks. This research has extensive significance for comprehending the intricate connections between the stock market and macroeconomic issues.

## Introduction

The Indian economy is ranked tenth in terms of nominal gross domestic product (GDP), and the International Monetary Fund (IMF) reports that it is the third largest economy in the world in terms of purchasing power parity (PPP). Kumar *et al.* (2023) have observed a significant increase in the quantity and volume of investments made in the Indian stock market both domestic and foreign investors in the past two decades. One of the key elements of an economy is the stock market. Its significance lies in its ability to propel a nation’s economy forward. The stock market serves as a gauge of the health of the economy and a vehicle for capital formation. An efficient economy can be inferred from the presence of a robust banking system and a stock market that is consistently increasing in value ( Kumari, 2011). The two primary functions of the stock markets are aiding in price discovery and offering liquidity. They facilitate firms in undertaking large-scale initiatives by enhancing the primary market. A strong stock market helps to stimulate the economy by facilitating efficient growth and saving, wise investment allocation, and the draw of foreign direct investment (FDI). Nevertheless, for the stock market to fulfil its vital role, it is imperative that it maintains a robust interaction with macroeconomic forces ( Tripathi, V., & Seth, R. 2014).

The impact of economic fundamentals on stock returns or prices has been a topic of much discussion among experts and scholars. The Efficient Market Hypothesis, initially introduced by Fama in 1970, asserts that in a market that operates efficiently, investors would not achieve exceptional profits as stock prices fully incorporate all pertinent information regarding fluctuations in macroeconomic variables. If the Efficient Market Hypothesis is assumed to be valid, any fluctuations in macroeconomic indicators should not exert a substantial influence on stock returns. Nevertheless, subsequent studies conducted by Fama and Schwert (1977), Nelson (1976), and other researchers have conducted a thorough assessment of the findings derived from the Efficient Market Hypothesis. These studies have verified that macroeconomic factors do indeed have an impact on stock prices, consequently influencing stock returns. A further theoretical contribution to the link between stock prices and the fundamentals of the economy is the Arbitrage Pricing Theory (APT) ( Ross, S.A. 1976; Chen *et al.,* 1986).

The link between macroeconomic variables and the stock market has been extensively studied in recent studies, building on previously established theoretical groundwork. The relationship between macroeconomic variables, stock market returns, and development in Ghana was assessed by Asravor, R. K., and Fonu, P. D. D. (2021), offering insights that might be useful for developing nations like India. Luo, C., Qu, Y., Su, Y., and Dong, L. (2024) examine the transmission of risks from crude oil markets to China’s financial market, emphasizing the importance of crude oil prices, which are equally relevant for the Indian stock market.

Previous research highlights the significance of GDP, inflation, crude oil prices, exchange rates, industrial production, and interest rates as key macroeconomic factors that impact the stock market. Fromentin, V. (2022), Bildirici *et al.* (2022), Ediriweera and Dissanayake (2022), and Katmas and Indarningsih (2022) documented the importance of macroeconomic forces in international markets over time, whereas Yadav *et al.* (2022), Shadab, A., and Sinha, A. K. (2022), Rao, V., and Jyotreddy, K. (2022), or Kumar *et al.,* (2023) provide evidence from the Indian market. The hypothesis that portfolio holder expectations about the future values of macroeconomic variables can impact stock prices and that these variables become risk factors when stocks are substituted for other assets in the portfolio is supported by the studies listed above. So, it is very important to look into how much macroeconomic indicator affect the stock markets.

## Review of literature

Many studies have looked at how the stock market is connected to macroeconomic elements. Previous studies indicate that macroeconomic variables and the stock market have an association. Fama (1981) sought to examine the link between stock returns, economic activity, inflation, and money in order to question preconceptions and offer new viewpoints on the dynamics of equities markets. While stock returns and real factors including output, capital expenditures, and the actual rate of return on capital show a positive link, the research results show a negative link between money growth rate and inflation.

Neifar *et al.* (2021) studied the relationship between macroeconomic indicators (currency rate, consumer price index, and interest rate) and the stock market in the United Kingdom. The ARDL model shows that there is a long-term symmetric link between macroeconomic data and the price of stocks. Conversely, the ECM representation’s results indicate that the UK stock price is rapidly advancing toward long-term equilibrium as a result of all of the macroeconomic variables that were assessed. According to Pole, H., and Cavusoglu, B. (2021), money supply and aggregate industrial output positively and significantly affect stock return volatility, whereas inflation and currency rates negatively affect it. This study used ARDL bonds to investigate the impact of macroeconomic variables on Nigerian stock market returns. Asravor, R. K., and Fonu, P. D. D. (2021) examined the connection between Ghana’s macroeconomic variables, stock market returns, and development revealed that interest rates and foreign direct investment were favourable to S.M. Development, but human capital, inflation, and money supply were awful. Using Jordan and Philips’ dynamic ARDL simulation model, Khan *et al.* (2021) studied exchange rate, gold, oil, and Shanghai stock exchange return effects. Although gold and oil prices positively affected return, the exchange rate negatively affected it. Chinese stock exchange rate fluctuations do not, in the long term, have a Granger-cause on stock returns, but they do have a substantial spillover effect, according to research by Cuestas and Tang (2021). Okorie *et al.* (2021) investigated the correlation between Nigerian inflation, currency rates, and stock market returns by employing copulas. It has been determined that there is a minor positive correlation between Nigerian inflation and stock market returns, as well as a positive association between Nigerian inflation and the exchange rate of the Nigerian Naira against the US dollar. Hung (2022c) investigates the dynamic connectedness between macroeconomic data and the Chinese stock market through wavelet coherence analysis. Short-term macroeconomic variables affect the stock market, whereas long-term stock gains improve macroeconomic conditions. Javangwe and Takawira (2022) examined the South African stock market and currency rates using ARDL. The stock market is found to be adversely affected by exchange rate fluctuations in the long term. Using ECM, Katmas and Indarningsih (2022) investigated at how inflation, interest rates, and foreign exchange rates impacted the Indonesian Sharia Stock Index (ISSI) and found that BI interest has a long-term effect on the index, exchange rates have both short- and long-term effects, and inflation has no effect. Economic growth variables and the time valve of money were the two components identified by Ediriweera and Dissanayake (2022) using factor analysis. The Time Value of Money component substantially affects the Colombo Stock Exchange, but Economic Growth has little effect. Bildirici *et al.* established a complex link between VIX investor mood, gold prices, currency rates, oil prices, and stock market returns in 2022. Nevertheless, the Turkish lira and energy prices have a significant influence on stock market returns. Fromentin (2022) employed the time-varying Granger causality framework to identify asymmetric bidirectional causation, which implies that the lead-lag relationship between macroeconomic variables and US stock price is subject to change over time.

Gay (2008) concluded that no significant link exists between exchange rates, oil prices, and stock market index prices in any BRIC country, so implying a weak form of market efficiency in Brazil, Russia, India, and China by using the Box-Jenkins ARIMA model to investigate the correlation between macroeconomic variables and stock market index prices in BRIC nations. Salisu *et al.* (2021) investigated the potential of stock return differentials to anticipate exchange rate fluctuations in the BRICS countries, thereby evaluating the validity of the uncovered equity parity (UEP) theory for these countries. For three BRICS countries—Brazil, India, and South Africa—the research found a positive association between stock return differentials and exchange rate returns; results for China and Russia were opposite. Hung (2022b) using the MGARCH-DCC and Wavelet Coherence Transform approaches investigated the temporal and frequency link between worldwide crude oil, gold prices, and financial markets in the BRICS countries. The BRICS stock and exchange markets have long-term influence on crude oil and gold variables; yet, in the immediate term, the international commodities market greatly influences the BRICS financial markets. Hung (2022a) investigated the dynamic relationship and volatility spillover effects between exchange rates and stock returns in five Central and Eastern European (CEE) countries (Hung Hungary, Poland, the Czech Republic, Romania, and Croatia) and identified bidirectional volatility spillovers between the two financial markets in Hungary, the Czech Republic, and Croatia during the pre-crisis period; a unidirectional spillover of volatility from the stock market to the foreign exchange market was observed. Atif *et al.* (2022) evaluated the correlation between stock returns, exchange rates, and crude oil in emerging markets. Panel Granger causality revealed that the relationship between stock returns and oil prices has grown stronger since COVID-19 was designated a pandemic. The oil market and currency rates are two other factors that have a beneficial impact on stock performance. Hashmi, S. M., and Chang, B. H. (2023) discovered the positive relationship between E7 stock market and macroeconomic variables. Lone *et al.* (2023) found that selected macroeconomic variables affect the stock markets of all BRICS nations except Brazil in the long term.

Pal, K., and Mittal, R. (2011) examined the relationship between Indian capital markets and macroeconomic indicators. The analysis found that inflation has a major impact on both the S&P CNX Nifty and the BSE Sensex, although only the former affects interest rates and the latter foreign currency rates. Kalra (2012) documented the Sensex and macroeconomic data link. The gold price, inflation rate, and foreign exchange rate have been regarded as the most important components in the development of Sensex forecast models. Singh (2014) found that while money supply and foreign investment favourably influence the Indian stock market, gold prices and currency values have a negative impact there. Gurloveleen and Bhatia (2015) discovered that the exchange rate and foreign institutional investors were significant factors after doing multiple regression analyses. Furthermore, there is no causal association between relevant factors and the average closing prices of BSE 500 manufacturing enterprises, according to the Granger causality test. Kotha and Bhawna (2016) found that money supply and inflation positively affect the Indian stock market return, whereas interest rates negatively affect it. Misra (2018) employed the Vector Error Correction Model (VECM) to analyse the BSE Sensex’s relationship with various macroeconomic variables, concluding that these variables, including money supply, inflation, interest rates, gold prices, foreign institutional investment, exchange rate, and the Index of Industrial Production, exhibited a long-term causal relationship with the Sensex. Mohanty *et al.* (2021) used the Auto Regressive Distributed Lag (ARDL) model to look into how macroeconomic factors affect stock market returns. While the unemployment rate and inflation have a negative effect, the Sensex is favourably influenced by the manufacturing to GDP ratio, debt to GDP ratio, US dollar, and GDP growth. Interest has a negative effect on share prices but a positive effect on consumer prices and industrial output, according to Syed (2021), who looked at how macroeconomic events affect Bombay Stock Exchange share prices. Bhattacharjee, A., and Das, J. (2021) employed the autoregressive distributed lag (ARDL) bounds testing approach and the pair-wise Granger causality test to examine the long- and short-term relationships between domestic macroeconomic variables and the equities market. The exchange rate, wholesale price index, and money supply lag exert a considerable short-term effect, while the money supply and foreign currency exchange rate significantly influence the Indian stock market in the long run. Yadav *et al.* (2022) identified a substantial relationship between macroeconomic factors and the Indian stock market price using Johansen cointegration and sought long-run equilibrium using VECM. Using the GARCH model, Shadab, A., and Sinha, A. K. (2022) found a positive link between exchange rate and BSE Sensex. Rao, V., and Jyotreddy, K. (2022) investigated the relationship between stock price and macroeconomic variables using the ARDL model and found that, in the long run, the relationship is negligible; but, in the short run, foreign institutional investment and inflation positively influence stock prices while exchange rate has negative influence. Kumar *et al.* (2023) used the NARDL model to find that crude oil prices positively affect the Indian stock market and negatively affect the exchange rate, but gold prices had no effect.

Though past studies have examined the relationship, opinions and clarity on how macroeconomic events influence the market are lacking. Previous studies yield contradicting findings; some revealed positive associations while others revealed negative or none at all. Bhattacharjee, A., and Das, J. (2021) discovered no correlation. Although Tripathi, V., and Seth, R. (2014) discovered that the Wholesale Price Index had a favourable effect on the Indian stock market. While Syed, A. A. (2021) found negative influence of interest rate, further Bhattacharjee, A., and Das, J. (2021) reported no association. Furthermore, displaying varying results is the gold price; Bhattacharjee, A., and Das, J. (2020) found negative influence and later in 2021 found no correlation. Studies on exchange rates yield inconsistent results with Ratanapakorn, O., and Sharma, S. C. (2007) noting positive influence while Syed, A. A. (2021) recorded negative impact. Different conclusions highlight the complexity of the link between macroeconomic factors and stock market.

It is necessary to offer unambiguous understanding on the link between macroeconomic variables and stock market. Few chosen variables have been used in past research at a time combined effect of all these variables is not investigated. Using ARDL bonds test, we have investigated the link between macroeconomic variables and stock market to close these discrepancies. Using monthly data from April 2009 to March 2023, the influence and most important macroeconomic factors influencing the stock market return in India are investigated and identified.

## Methods

In this paper we have used monthly data from April 2009 to March 2023 to study the impact of Wholesale price index (WPI), Consumer price index (CPI), Index of Industrial Production (IIP), Gross Domestic Product (GDP), Foreign Exchange Reserve (FER), Exchange Rate (ER), Silver Price (SP), Gold Prices (GP), Brent Crude Oil Prices (COP), Gross Fiscal Deficit (GFD), Value of Imports (IMP), Value of Exports (EXP), Foreign Institutional Investors (FII), Repo rate (REPO), Interest Rate (Long Term 10 yr) (INT), Call Money Rate (CMR), Balance of Payment (BOP), Broad Money (M3) on National Stock Exchange returns (RNSE) ( Table 1). The autoregressive distributed lag (ARDL) bounds testing approach is used to investigate long-term correlations between variables. Pesaran *et al*. created it in 2001. Time series that are neither stationary nor have a fixed order of integration can be fitted using the ARDL model, which is based on ordinary least squares (OLS).

**Figure 1.**
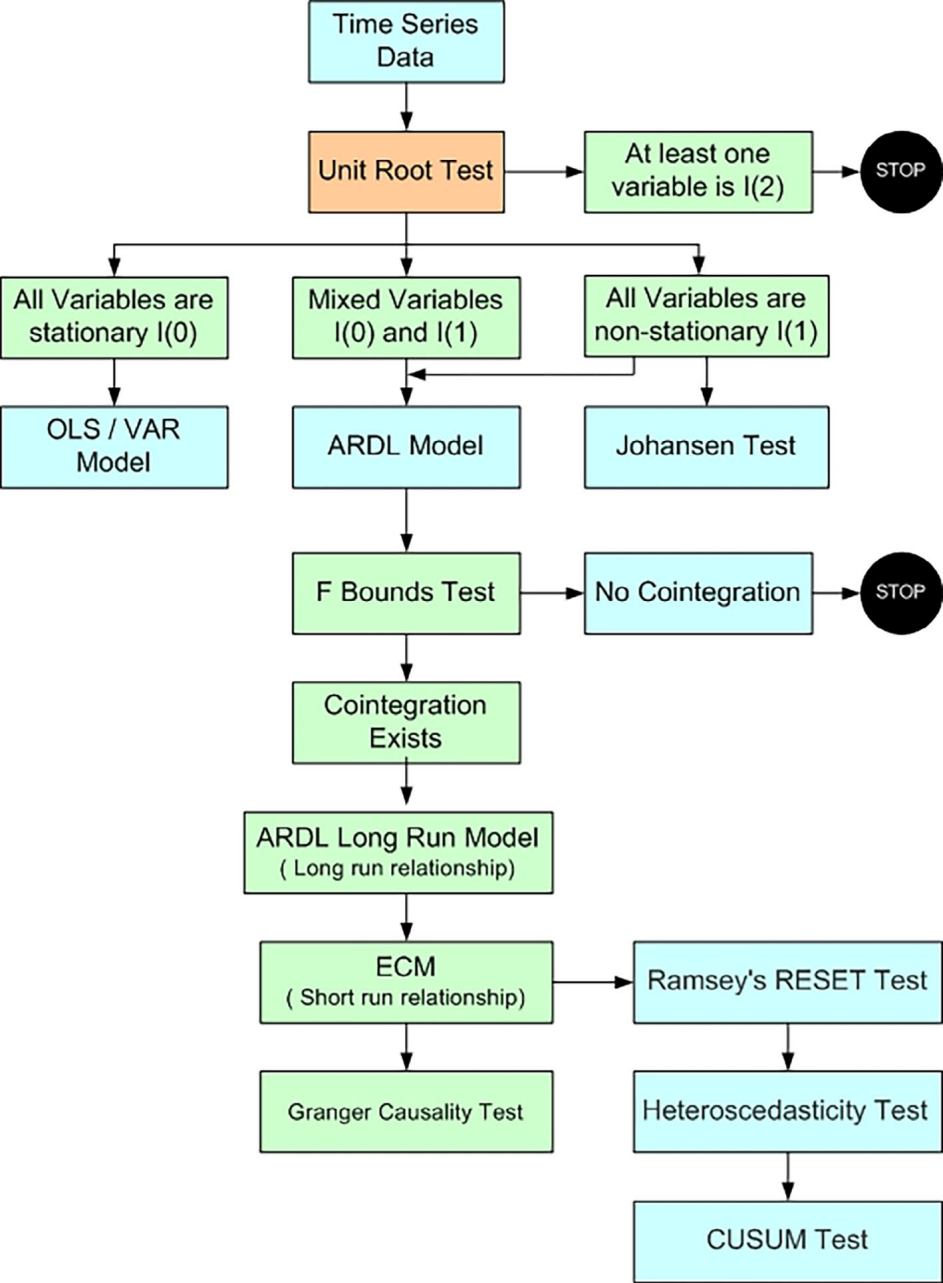
Methodological Process (Author’s compilation).

**Table 1.** Variables of the study.

S.No	Variables	Symbol	Data Source	Unit of measurement
1	Nifty50	NSE	Investing.com	Return
2	Wholesale price index	WPI	Investing.com	Annual growth rate (%)
3	Consumer price index	CPI	Investing.com	Annual growth rate (%)
4	Index of Industrial Production	IIP	Investing.com	% change previous period
5	Gross Domestic Product	GDP	Investing.com	% change previous period
6	Foreign Exchange Reserve	FER	RBI	INR (Cr)
7	Exchange Rate	ER	Investing.com	INR/USD
8	Silver Price	SP	Yahoo Finance	USD
9	Gold Prices	GP	Investing.com	INR
10	Brent Crude Oil Prices	COP	U.S. Energy Information Administration (EIA)	USD/Barrel
11	Gross Fiscal Deficit	GFD	RBI	INR (Cr)
12	Value of Imports	IMP	IMF	USD (Millions)
13	Value of Exports	EXP	IMF	USD (Millions)
14	Foreign Institutional Investors	FII	Central Depository Services (India) Limited	INR (Cr)
15	Repo rate	REPO	bankbazaar.com	Percentage
16	Interest Rate (Long Term 10 yr)	INT	OECD	% per annum
17	Call Money Rate	CMR	RBI	% P.a.
18	Balance of Payment	BOP	RBI	INR (cr)
19	Money Supply	M2	Federal Reserve Economic Data (FRED)	INR

The current study applied Pesaran *et al.* (2001) Autoregressive Distributed Lag (ARDL) model. This method is applied since it provides strong findings when the dataset is small and the variables show a mix of integration orders, that is, I(0) and I(1) variables. We must first make sure none of the variables are stationary at second difference before using the ARDL model. We investigate at which level variables are stationary using the Augmented Dickey-Fuller ( Phillips & Perron, 1988). The null hypothesis posits the existence of a unit root, while the alternative hypothesis suggests stationarity.

The augmented Dickey-Fuller test conducts the regression analysis described below.

$$y_{t}=a+\beta_{t}+\gamma y_{t-1}+\sum_{i=1}^{p}\delta_{i}∆y_{t-i}+e_{t}$$ (1)

y(t-1) = lag 1 of time series and delta Y(t-1) = first difference of the series at time (t-1)

Fundamentally, it has a similar null hypothesis as the unit root test. That is, the coefficient of Y(t-1) is 1, implying the presence of a unit root. If not rejected, the series is taken to be non-stationary.

ADF test results assist ARDL application to investigate co-integration among the variables. If the variables are stationery at level Johansen cointegration test is appropriate to investigate the long-term relationship among the variables. The equation used in the present study to specify the ARDL model is as follows:

$$∆y_{t}=\alpha_{0}+\sum_{i=1}^{p}\alpha_{1}∆y_{t-i}+\sum_{i=0}^{q}\alpha_{2}∆x_{t-i}+\delta_{1}y_{t-1}+\delta_{2}x_{t-1}+\varepsilon_{t}$$ (2)

Where $x_{t}$ vector of regressors, $y_{t}$ dependent variable; $\alpha_{0}$ intercept; Δ is the first difference operator; $\alpha_{i}$ represent short-run coefficient; $\delta_{i}$ represent long-run coefficient; *p* and *q* represent restricted lags and $\varepsilon_{t}$ shows error term. The estimated F-statistic indicates that the variables are cointegrated, therefore assuming a long-term link between them, if it surpasses the upper bound critical value. The test is said to be inconclusive, on the other hand, if the F-statistic goes below the upper bound critical value but stays above the lower bound critical value. It follows from an F-statistic smaller than the lower bound critical value that the variables are not cointegrated. Should the ARDL limits test verify cointegration, the long-run coefficients and short-run parameters including the error correction term are next to be estimated ( Shrestha, M. B. & Bhatta, G. R., 2018). We then evaluate the model’s dependability with several diagnostic tests. The functional form of the model is checked using Ramsey’s RESET test; heteroscedasticity is tested using Breusch-Pagan–Godfrey test; serial correlation between the residuals is tested using Breusch-Godfrey serial correlation lag range multiplier (LM); and CUSUM test checks the stability of the coefficients. We investigated the direction of short-term causality using the pairwise Granger causality test ( Granger, C. W., 1969).

## Analysis and Discussion

time series analysis, ascertaining the stationarity status is a crucial step. This study employed the Augmented Dickey-Fuller test to ascertain the stationarity status ( Table 2). The ADF test results demonstrate that LnIIP, LnGDP, and LnFII are stationary at level, but the remaining variables in the study are stationary at first difference. At the level, the null hypothesis that the series possesses a unit root is rejected for LnIIP, LnGDP, and LnFII, while it cannot be rejected for the remaining variables in the study. Upon the initial differencing, all non-stationary series attain stationarity.

**Table 2.** Unit Root Test (ADF).

Null hypothesis: Series has a unit root					
	Level		First difference		Order of integration
	t-Statistic	p-Value	t-Statistic	p-Value	
LnNSE	0.65331	0.8540	10.66795	0.0000	I(1)
LnWPI	2.86468	0.0518	4.34557	0.0005	I(1)
LnSP	1.85264	0.3541	6.39621	0.0000	I(1)
LnREPO	0.12702	0.9669	4.35494	0.0005	I(1)
LnM3	2.66303	0.0827	2.93487	0.0436	I(1)
LnINT	1.59587	0.4825	4.88953	0.0001	I(1)
LnIMP	2.92610	0.0445	8.53574	0.0000	I(1)
LnIIP	6.05275	0.0000			I(0)
LnGP	2.25023	0.1896	6.64226	0.0000	I(1)
LnGFD	2.73658	0.2541	6.39573	0.0000	I(1)
LnGDP	4.03958	0.0016			I(0)
LnFII	9.05679	0.0000			I(0)
LnFER	1.50637	0.5281	3.54933	0.0079	I(1)
LnEXP	2.78522	0.0625	7.85539	0.0000	I(1)
LnER	1.64973	0.4550	5.14721	0.0000	I(1)
LnCPI	2.02833	0.2746	9.32421	0.0000	I(1)
LnCOP	1.58457	0.4882	6.58330	0.0000	I(1)
LnCMR	1.55676	0.5024	6.17225	0.0000	I(1)
LnBOP	4.32833	0.0006			I(0)

Subsequent to establishing the stationarity of the variables, we employed the ARDL bounds test to investigate the presence of a long-run relationship among the variables.

Table 3 presents the findings of the Autoregressive Distributed Lag Bounds Test for cointegration, indicating a significant long-term equilibrium link among the variables. The F-statistic of 7.117339, obtained from the test, exceeds the upper bound critical values at all conventional significance levels: 2.77 at 10%, 3.04 at 5%, 3.28 at 2.50%, and 3.61 at 1%. The null hypothesis of no cointegration is rejected, confirming a long-term link between the examined variables.

**Table 3.** ARDL Bounds Test.

Test statistic	Value	Significance level	I(0)	I(1)
F-statistic	7.117339	10%	1.76	2.77
		5%	1.98	3.04
		2.50%	2.18	3.28
		1%	2.41	3.61

The long-run ARDL results shown in Table 4 show that money supply (M3), GDP, foreign institutional investment (FII), exports (EXP), exchange rates (ER), and crude oil prices (COP) has statistically significant effect on the stock price. LnM3, LnGDP, LnFII, andLnCOP have positive coefficients indicating that stock price rises follow from changes in these variables. Should M3, GDP, FII, and COP rise by 1% stock price, the respective increases will be 1.37%, 0.12%, 0.09% and 0.30%. By contrast, LnEXP and LnER have negative coefficients, meaning a negative effect on stock price. Regarding the money supply, the outcomes contradict Asravor, R. K., & Fonu, P. D. D. (2021) and line Pole, H., and Cavusoglu, B. (2021). For GDP results match Parab, N., and Reddy, Y. V. (2020) and Abu-Libdeh, H., and Harasheh, M. (2011). Further crude oil price results contradict ( Abuoliem *et al.*, 2019) but support Jain, A., and Biswal, P. C. (2016).

**Table 4.** Long Run Form of ARDL.

Model (2, 0, 1, 0, 0, 0, 1, 1, 1, 0, 0, 0, 1, 0, 1, 1, 0, 0, 0)				
Variable	Coefficient	Std. Error	t-Statistic	Prob.
LnWPI	0.022254	0.037985	0.585859	0.5589
LnSP	-0.306743	0.180919	-1.69547	0.0922***
LnREPO	-0.718907	0.377801	-1.902871	0.0591***
LnM3	1.37184	0.470784	2.913945	0.0042*
LnINT	0.499693	0.350984	1.423692	0.1568
LnIMP	-0.030099	0.203991	-0.147549	0.8829
LnIIP	0.06365	0.052582	1.21051	0.2282
LnGP	0.329263	0.333996	0.985829	0.3259
LnGFD	-0.002413	0.011244	-0.214605	0.8304
LnGDP	0.127941	0.05071	2.522993	0.0128**
LnFII	0.099797	0.024157	4.1312	0.0001*
LnFER	0.031715	0.454223	0.069822	0.9444
LnEXP	-0.590043	0.295944	-1.993767	0.0481**
LnER	-1.24941	0.552302	-2.262188	0.0252**
LnCPI	-0.109402	0.056085	-1.950641	0.0531***
LnCOP	0.302682	0.119182	2.539658	0.0122**
LnCMR	0.131846	0.194867	0.676594	0.4998
LnBOP	-0.011307	0.008135	-1.389949	0.1668
C	-12.52415	3.264108	-3.836927	0.0002

EC = LNNSE - (0.0223*LNWPI -0.3067*LNSP -0.7189*LNREPO + 1.3718*LNM3 + 0.4997*LNINT -0.0301*LNIMP + 0.0637*LNIIP + 0.3293*LNGP -0.0024*LNGFD + 0.1279*LNGDP + 0.0998*LNFII + 0.0317*LNFER -0.5900*LNEXP -1.2494*LNER -0.1094*LNCPI + 0.3027*LNCOP + 0.1318*LNCMR -0.0113*LNBOP - 12.5241).

Note: *, ** and *** Indicate significance at the 1%, 5% and 10% level, respectively.

With coefficients of -0.306743, -0.7189 and -0.1094, respectively, the Silver Price (SP), repo rate (REPO) and consumer price index (CPI) show negative associations, significant at the 10% level. Other variables, such as the wholesale price index, long-term interest rates, imports, industrial production index, gold price, gross fiscal deficit, foreign currency reserves, call money rate, and balance of payments, do not have statistically significant long-term effects. The model’s constant term is significant, as indicated by its coefficient of -12.5241. These findings emphasize the significant influences on the future behaviour of the dependent variable, which include money supply, GDP, foreign institutional investment, exports, currency rates, and crude oil prices.

The error correction model results ( Table 5) reveal the short-term dynamics and the pace of adjustment towards the long-run equilibrium. The highly significant and negative error correction coefficient implies that approximately 22.74% of the deviation from the long-run equilibrium is rectified within one month. Lag of stock price, value of import, and exchange rate has significant negative impact on stock price. Impact of foreign exchange reserve is positive and significant. Furthermore, the model demonstrates strong explanatory power, with an R-squared value of 0.677981, highlighting its robustness in capturing the interrelationships among the variables.

**Table 5.** Error Correction form.

Variable	Coefficient	Std. Error	t-Statistic	Prob.
D (LnNSE(-1))	-0.132218	0.048813	-2.70869	0.0076**
D (LnSP)	-0.000665	0.031748	-0.020939	0.9833
D (LnIMP)	-0.105707	0.036631	-2.885751	0.0045**
D (LnIIP)	0.000404	0.006163	0.065519	0.9479
D (LnGP)	-0.059503	0.058602	-1.015377	0.3117
D (LnFER)	0.385728	0.129725	2.973437	0.0035**
D (LnER)	-1.37868	0.13982	-9.860403	0.0000*
D (LnCPI)	0.020965	0.015267	1.373214	0.1719
CointEq(-1)*	-0.227427	0.017871	-12.72577	0.0000*
R-squared	0.677981			

Note: *, ** and *** Indicate significance at the 1%, 5% and 10% level, respectively.

**Figure 2.**
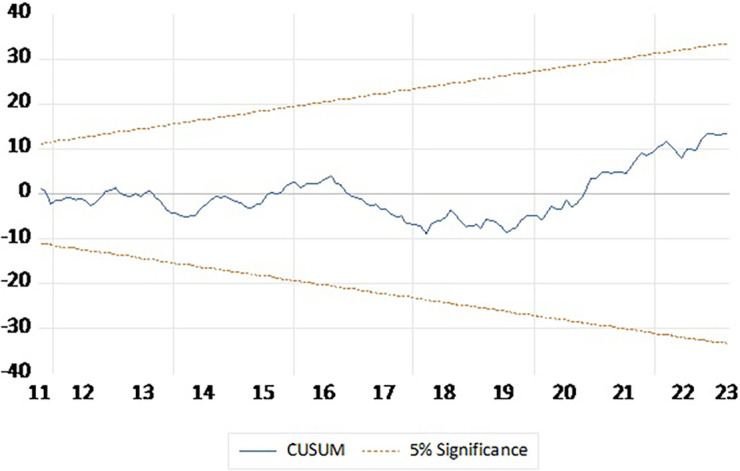
CUSUM plot.

To check reliability of the short run estimates Ramsey RESET and Breusch–Pagan–Godfrey Heteroskedasticity Test to check the reliability the short run estimates and CUSUM plot to test stability of the model. The Ramsey RESET test yields an F-statistic of 0.075087 with a p-value of 0.7845, indicating no evidence of model misspecification. The Breusch-Pagan-Godfrey test for heteroskedasticity provided an F-statistic of 0.953374 with a p-value of 0.5367, suggesting the residuals exhibit homoscedasticity ( Table 6). CUSUM plot presented in Figure 2 indicate that the model is stable and outcomes are reliable.

**Table 6.** Residual diagnostic test.

	Test statistics	p-Value
Ramsey RESET Test, *F*-statistic	0.075087	0.7845
Heteroskedasticity Test: Breusch-Pagan-Godfrey, *F*-statistic	0.953374	0.5367

Granger causality examined the significance of the coefficients of lagged $y_{t}$ , which are used as the explanatory variables for $x_{t}$ in the regression context. For a simple bivariate model, one can test the following equation:

$$x_{t}=\alpha_{0}+\sum_{i=1}^{p}\alpha_{i}y_{t-i}+\sum_{j-1}^{q}\beta_{j}x_{t-j}+u_{t}$$ (3)

$$y_{t}=\alpha_{0}+\sum_{i=1}^{p}\beta_{i}x_{t-i}+\sum_{j-1}^{q}\alpha_{j}y_{t-j}+\varepsilon_{t}$$ (4)

Where Ho: *y* does not Granger causes *x* in the first regression equation and *x* does not Granger causes *y* in the second regression equation.

Results of pairwise Granger causality test ( Table 7) Indicate that there is bidirectional causality between IMP and NSE, FII and NSE, FER and NSE, COP and NSE signifying the presence of feedback mechanism between the variables. Unidirectional causality is identified between WIP and NSE, SP and NSE, GDP and NSE, EXP and NSE, ER and NSE. Result indicates No causality between REPO and NSE, M3 and NSE, INT and NSE, IIP and NSE, GP and NSE, GFD and NSE, CPI and NSE, CMR and NSE, BOP and NSE. Pairwise Granger Causality Test findings suggest that IMP, FII, FER, COP, WIP, SP, GDP, EXP and ER are helpful in stock price prediction in Indian stock market.

**Table 7.** Pairwise Granger Causality Tests.

Pair	Null Hypothesis:	F-Statistic	Prob.	Result
WIP and NSE	LNWPI does not Granger Cause LNNSE	0.59491	0.6192	Unidirectional causality
	LNNSE does not Granger Cause LNWPI	2.72148	0.0463**	
SP and NSE	LNSP does not Granger Cause LNNSE	2.05970	0.1078	Unidirectional causality
	LNNSE does not Granger Cause LNSP	3.03723	0.0308**	
REPO and NSE	LNREPO does not Granger Cause LNNSE	1.74601	0.1598	No causality
	LNNSE does not Granger Cause LNREPO	0.76984	0.5125	
M3 and NSE	LNM3 does not Granger Cause LNNSE	1.68342	0.1728	No causality
	LNNSE does not Granger Cause LNM3	0.50557	0.6790	
INT and NSE	LNINT does not Granger Cause LNNSE	1.15080	0.3305	No causality
	LNNSE does not Granger Cause LNINT	1.45463	0.2291	
IMP and NSE	LNIMP does not Granger Cause LNNSE	2.61472	0.0531***	Bidirectional causality
	LNNSE does not Granger Cause LNIMP	14.41820	0.0000*	
IIP and NSE	LNIIP does not Granger Cause LNNSE	0.59025	0.6223	No causality
	LNNSE does not Granger Cause LNIIP	0.57354	0.6332	
GP and NSE	LNGP does not Granger Cause LNNSE	0.93629	0.4247	No causality
	LNNSE does not Granger Cause LNGP	0.75005	0.5239	
GFD and NSE	LNGFD does not Granger Cause LNNSE	0.01070	0.9985	No causality
	LNNSE does not Granger Cause LNGFD	0.27888	0.8406	
GDP and NSE	LNGDP does not Granger Cause LNNSE	1.76157	0.1567	Unidirectional causality
	LNNSE does not Granger Cause LNGDP	8.30001	0.0000*	
FII and NSE	LNFII does not Granger Cause LNNSE	2.50823	0.0609***	Bidirectional causality
	LNNSE does not Granger Cause LNFII	2.99270	0.0327**	
FER and NSE	LNFER does not Granger Cause LNNSE	4.47503	0.0048*	Bidirectional causality
	LNNSE does not Granger Cause LNFER	2.29608	0.0798***	
EXP and NSE	LNEXP does not Granger Cause LNNSE	0.75034	0.5237	Unidirectional causality
	LNNSE does not Granger Cause LNEXP	10.99250	0.0000*	
ER and NSE	LNER does not Granger Cause LNNSE	2.13205	0.0983***	Unidirectional causality
	LNNSE does not Granger Cause LNER	1.99846	0.1164	
CPI and NSE	LNCPI does not Granger Cause LNNSE	0.87646	0.4547	No causality
	LNNSE does not Granger Cause LNCPI	0.56382	0.6397	
COP and NSE	LNCOP does not Granger Cause LNNSE	2.18345	0.0921***	Bidirectional causality
	LNNSE does not Granger Cause LNCOP	7.28678	0.0001*	
CMR and NSE	LNCMR does not Granger Cause LNNSE	1.14049	0.3346	No causality
	LNNSE does not Granger Cause LNCMR	0.74170	0.5287	
BOP and NSE	LNBOP does not Granger Cause LNNSE	0.09832	0.9609	No causality
	LNNSE does not Granger Cause LNBOP	1.23910	0.2974	

Note: *, ** and *** Indicate significance at the 1%, 5% and 10% level, respectively.

## Conclusion and Recommendations

The ARDL bonds test validates the presence of an enduring connection between the variables. The ARDL model demonstrates that over a long period of time, the money supply (M3), gross domestic product (GDP), foreign institutional investment (FII), and crude oil prices (COP) have a substantial positive influence on the stock price. Conversely, exchange rates (ER) and exports (EXP) have a major negative impact. In the near term, there is a large negative correlation between the lag of stock price, value of imports, and exchange rate with stock price. However, the influence of foreign exchange reserves on stock price is positive. The diagnostic tests performed on the residuals to confirm the accuracy and consistency of the short-run parameters indicated no signs of serial correlation, heteroscedasticity, or misspecification. The findings of the paired Granger causality test indicate that the domestic macroeconomic variables have predictive value for stock prices in the Indian stock market. Therefore, it is important to closely watch these variables.

The study’s findings suggest that macroeconomic variables have an impact on the Indian stock market both in the short term and the long term. Hence, it is imperative for investors and portfolio managers to regularly monitor the prevailing macroeconomic conditions in order to optimize their returns and mitigate potential risks. Furthermore, the finding has significant policy consequences. A rise in the money supply has a beneficial impact on the Indian stock market by enhancing a firm’s capacity to produce and borrow. The Reserve Bank of India (RBI) should strive to maintain an optimal level of money supply by utilizing open market operations and monetary policy. This is crucial because an excessive amount of money in circulation can lead to inflation, which has a detrimental impact on the Indian stock market. Furthermore, the exchange rate has a detrimental effect on the Indian stock market. The Reserve Bank of India (RBI) should implement measures to mitigate fluctuations in the exchange rate. The Indian government should formulate policies to bolster the export sector and facilitate the import of diverse items. The Atmanirbhar Bharat Abhiyan and Make in India initiatives are remarkable endeavours in this context; yet, the effectiveness of these initiatives relies on meticulous execution. The positive impact of GDP underscores the significance of continuous economic expansion, necessitating policies that focus on improving productivity, developing infrastructure, and creating a favourable business environment. Enhancing the inflow of Foreign Direct Investment (FDI) and Foreign Institutional Investment (FII) can significantly enhance economic performance. This necessitates the establishment of a stable and conducive regulatory framework that is attractive to investors.

## Ethics and consent

Ethical approval and consent were not required.

## Data Availability

The underlying data related to the paper are available in figshare with the following citation and DOI: https://doi.org/10.6084/m9.figshare.27044473 ( Sanjay Singh Chauhan *et al.*, 2024) A causality investigation into stock prices and macroeconomic indicators in the Indian stock market © 2024 by Sanjay Singh Chauhan, Dr. Pradeep Suri, Dr. Debapriyo Nag, Dr. Farman Ali is licensed under CC BY 4.0 Attribution 4.0 International.
