# Supplementary material for: A causality investigation into stock prices and macroeconomic indicators in the Indian stock market
Source: F1000Res. 2025 Jun 3;13:1299. Originally published 2024 Oct 30. [Version 3] doi: 10.12688/f1000research.157041.3 (PMC12144490; doi:10.12688/f1000research.157041.3)
Supplement: Peer review history

# Reviewer report: Reviewer response for version 3

**DOI:** 10.5256/f1000research.183120.r389494

**Published:** 2025-06-06

**Authors:** Hazman Samsudin

**Affiliations:** 1 The Universiti Malaysia Terengganu, Terengganu, Malaysia

I now recommend the manuscript to be indexed.

Is the work clearly and accurately presented and does it cite the current literature?

Partly

If applicable, is the statistical analysis and its interpretation appropriate?

Partly

Are all the source data underlying the results available to ensure full reproducibility?

Yes

Is the study design appropriate and is the work technically sound?

Partly

Are the conclusions drawn adequately supported by the results?

Yes

Are sufficient details of methods and analysis provided to allow replication by others?

Partly

Reviewer Expertise:

Finance and International economics, econometrics, development economics

I confirm that I have read this submission and believe that I have an appropriate level of expertise to confirm that it is of an acceptable scientific standard.

# Reviewer report: Reviewer response for version 3

**DOI:** 10.5256/f1000research.183120.r389495

**Published:** 2025-06-06

**Authors:** Aamir Aijaz Syed

**Affiliations:** 1 National Institute of Technology, Allahabad, India

I am satisfied with the revised version and approve the version shared.

Is the work clearly and accurately presented and does it cite the current literature?

Partly

If applicable, is the statistical analysis and its interpretation appropriate?

Partly

Are all the source data underlying the results available to ensure full reproducibility?

Yes

Is the study design appropriate and is the work technically sound?

Yes

Are the conclusions drawn adequately supported by the results?

Yes

Are sufficient details of methods and analysis provided to allow replication by others?

Yes

Reviewer Expertise:

Finance and Economics

I confirm that I have read this submission and believe that I have an appropriate level of expertise to confirm that it is of an acceptable scientific standard.

# Reviewer report: Reviewer response for version 2

**DOI:** 10.5256/f1000research.179731.r382758

**Published:** 2025-05-15

**Authors:** Aamir Aijaz Syed

**Affiliations:** 1 National Institute of Technology, Allahabad, India

The author should include a clear theoretical contribution in the introduction section. 

 Literature review is adequate however in my opinion author is suggested to include comprehensive literature gap and how the present study fill this gap.

 The author should highlight the reason for employing the data from 2009 to 2023. 

 Author can add few recent studies:

 Company-specific antecedents to stock returns: a statistical investigation with special reference of the NIFTY 50. *(refer 1 )*

 Methodology is appropriate however I recommend the author should add few study strengthening the rational for employing ARDL method.

 Author can refer the following studies: 

 Investigating the impact of the first and second waves of the COVID-19 pandemic on the Indian stock and commodity markets: An ARDL analysis of gold, oil, and stock market prices. *(refer 2 )*.

 The author can strengthen the outcome using empirical literature which is missing

 Finally, policy specific recommendations need to be added. 

 Best Wishes

Is the work clearly and accurately presented and does it cite the current literature?

Partly

If applicable, is the statistical analysis and its interpretation appropriate?

Partly

Are all the source data underlying the results available to ensure full reproducibility?

Yes

Is the study design appropriate and is the work technically sound?

Yes

Are the conclusions drawn adequately supported by the results?

Yes

Are sufficient details of methods and analysis provided to allow replication by others?

Yes

Reviewer Expertise:

Finance and Economics

I confirm that I have read this submission and believe that I have an appropriate level of expertise to confirm that it is of an acceptable scientific standard, however I have significant reservations, as outlined above.

## References

[1] Goel, S., Yadav, N., Wani, N. U. H., & Syed, A. A. (2025). Company-specific antecedents to stock returns: a statistical investigation with special reference of the NIFTY 50. International Journal of Diplomacy and Economy, 11(2), 105-123.

[2] Syed, A., Tripathi, R., & Dewan, J. (2021). Investigating the Impact of COVID-19 Pandemic First and Second Wave on the Indian Stock and Commodity Market: An ARDL Analysis of Gold, Oil and Stock Market Price. Syed A. A, Tripathi. R, Dewan Jyoti. Investigating the impact of COVID, 19, 8-21.

# Reviewer report: Reviewer response for version 1

**DOI:** 10.5256/f1000research.172441.r369212

**Published:** 2025-03-25

**Authors:** Hazman Samsudin

**Affiliations:** 1 The Universiti Malaysia Terengganu, Terengganu, Malaysia

1. In the introduction, the author should address why an investigation on stock market is important. Perhaps the author should add some evidence on stock market cause a volatility. this have been documented such as in Hazman (2016)(refer 4),

 Dai et al., (2020)(refer 3)  and Dhingra et al., (2024)(refer 2). Please cite these articles and highlights why it is important to study on stock market.

 2. Literature review is fine but can be improve by writing it in the manner of discussions rather than just a list of literature.

 3. The method is a bit confusing. The title said a causality investigation and in result the author did mention about Granger causality, but the method used is ARDL. Please note that ARDL is cointegration test and not causality testing. Causality test is more suitable with Granger test and in the case of mixed stationarity as suggested by this study, Toda-Yamamoto causality testing seems more feasible and suitable. So this study investigating causality relationship or cointegration among the variables? please clarify and make it clear. and also are the data is in original form or have been log?

 4. The discussion on the results also a bit shallow. please elaborate more. I think it would be beneficial to cite this recent article in 2025 also- refer 1

Is the work clearly and accurately presented and does it cite the current literature?

Partly

If applicable, is the statistical analysis and its interpretation appropriate?

Partly

Are all the source data underlying the results available to ensure full reproducibility?

Yes

Is the study design appropriate and is the work technically sound?

Partly

Are the conclusions drawn adequately supported by the results?

Yes

Are sufficient details of methods and analysis provided to allow replication by others?

Partly

Reviewer Expertise:

Finance and International economics, econometrics, development economics

I confirm that I have read this submission and believe that I have an appropriate level of expertise to confirm that it is of an acceptable scientific standard, however I have significant reservations, as outlined above.

## References

[1] : The Dynamic Response of the Green Stock Market to External Economic Policy Uncertainty: The Case of Indonesia. *International Journal of Energy Economics and Policy* .2025;15(2) : 10.32479/ijeep.18254 360-369 10.32479/ijeep.18254

[2] : Stock market volatility: a systematic review. *Journal of Modelling in Management* .2024;19(3) : 10.1108/JM2-04-2023-0080 925-952 10.1108/JM2-04-2023-0080

[3] : Efficient predictability of stock return volatility: The role of stock market implied volatility. *The North American Journal of Economics and Finance* .2020;52: 10.1016/j.najef.2020.101174 10.1016/j.najef.2020.101174

[4] Samsudin, Hazman & Nawawi, Mohd Nasir & Abdul Halim, Zairihan & Md Said, Ahmad Syahmi, 2018. "Financial Performance Evaluation of Islamic Banking System: A Comparative Study among Malaysiaâ€™s Banks," Jurnal Ekonomi Malaysia, Faculty of Economics and Business, Universiti Kebangsaan Malaysia, vol. 52(2), pages 137-147.

# Reviewer report: Reviewer response for version 1

**DOI:** 10.5256/f1000research.172441.r368214

**Published:** 2025-03-06

**Authors:** Alireza Askarzadeh

**Affiliations:** 1 Old Dominion University, Norfolk, Virginia, USA

**Summary of the Paper**

 The paper investigates the causal relationship between macroeconomic indicators and stock prices in the Indian stock market using the ARDL bounds testing approach and Granger causality tests. The study analyzes data from April 2009 to March 2023, examining the long-run and short-run effects of variables such as money supply, foreign institutional investment (FII), crude oil prices, exchange rates, and GDP on stock market returns. The findings highlight significant predictive relationships, with policy implications for investors and regulators.

 While the paper presents a well-structured empirical analysis, there are several areas for improvement, particularly regarding methodological justification, causality interpretation, and the issue of endogeneity.

**Major Concerns**

**1. Endogeneity Issues (Reverse Causality & Omitted Variable Bias)**

 The study does not address potential endogeneity concerns, which are critical in empirical finance research. The relationship between macroeconomic indicators and stock prices is likely **bidirectional**:

- **Reverse Causality:** Stock prices themselves influence macroeconomic variables such as exchange rates and FII inflows, making it difficult to establish one-way causation.
- **Omitted Variable Bias:** External factors such as global market trends, investor sentiment, or monetary policy shocks could simultaneously affect both stock prices and macroeconomic indicators.

**Recommendation:** The authors should acknowledge this limitation and consider addressing it through:

- Instrumental Variable (IV) methods (e.g., lagged macroeconomic indicators or global oil price shocks).
- Structural Vector Autoregression (SVAR) models to capture dynamic interactions.
- Event study approaches if identifiable policy shocks exist.

Without addressing these concerns, the causal claims remain **associational rather than truly causal**.

**2. Justification of Model Choice**

 The use of the ARDL model is reasonable, but the authors should provide a stronger justification for selecting it over other alternatives like VAR or VECM. Specifically:

**3. Interpretation of Causality**

 The Granger causality test does not establish true causation but only predictive relationships. This should be made explicit in the discussion.

**4. Expanding Literature Comparison**

 The paper would benefit from a stronger comparison with similar studies in emerging markets. Studies from China, Brazil, and South Africa have examined similar relationships and should be referenced. This would help position the paper’s findings within a broader research context.

**5. Strengthening Policy Implications**

 The policy recommendations are insightful but remain broad. The authors should:

**Minor Concerns**

- **Presentation of Results**: Including impulse response functions or time-series graphs would improve the clarity of findings.
- **Significance Levels in Tables**: The authors should explicitly report significance levels (p-values) for each coefficient in the ARDL model.
- **Limitations Section**: A dedicated discussion of omitted variables, structural economic shifts (e.g., COVID-19), and potential model improvements should be included.

**Conclusion**

 This study makes an important contribution to understanding the interaction between macroeconomic indicators and stock prices in India. However, several methodological concerns—particularly regarding endogeneity, causality interpretation, and robustness—should be addressed to strengthen the paper’s scientific rigor.

**Recommendation:** **Revision**

 By incorporating these suggestions, the paper will provide a more compelling and reliable contribution to the literature on stock market-macroeconomic interactions.

 Sincerely,

**Alireza Askarzadeh**

**Finance Department**

**Old Dominion University**

**Norfolk, VA, USA**

Is the work clearly and accurately presented and does it cite the current literature?

Yes

If applicable, is the statistical analysis and its interpretation appropriate?

Partly

Are all the source data underlying the results available to ensure full reproducibility?

Partly

Is the study design appropriate and is the work technically sound?

Partly

Are the conclusions drawn adequately supported by the results?

Yes

Are sufficient details of methods and analysis provided to allow replication by others?

Yes

Reviewer Expertise:

Corporate finance, Investments

I confirm that I have read this submission and believe that I have an appropriate level of expertise to confirm that it is of an acceptable scientific standard, however I have significant reservations, as outlined above.

# Response

**Published:** 2025-03-21

**Authors:** Sanjay Chauhan

Respected Sir,

 I have incorporated most of your suggestions in the revised version. New version includes Impulse response function, limitations and P- Value in ARDL model. Further, I have also expanded the literature review for comparison as suggested.

 Thanks and Regards

 Sanjay Singh Chauhan

# Reviewer report: Reviewer response for version 1

**DOI:** 10.5256/f1000research.172441.r337233

**Published:** 2024-11-07

**Authors:** Baranidharan Subburayan

**Affiliations:** 1 Christ University, Bangalore, Karnataka, India

Your analysis provides a strong foundation, examining both short-term and long-term impacts of macroeconomic variables on the Indian stock market. The findings hold significance for investors and policymakers, and you present a clear framework for policy recommendations. However, to further enhance the rigour, clarity, and scientific soundness of your study, consider the following constructive suggestions

 1. Clarify the Methodological Approach

 While using the ARDL model is appropriate, it would be beneficial to justify why this model was selected over other econometric techniques, especially for readers less familiar with time-series methodologies. Could you briefly discuss why ARDL is suitable for analyzing the relationships between non-stationary variables?

 The diagnostic tests ensure the robustness of the model, yet further clarification on which specific tests (e.g., Breusch-Godfrey for serial correlation or Breusch-Pagan for heteroscedasticity) were used would enhance transparency. Detail the results and thresholds applied to interpret the presence or absence of statistical issues.

 2. Strengthen the Discussion on Causality and Interpretation

 While the Granger causality test is a useful tool for examining predictiveness, it does not confirm causation. A brief statement on this limitation would enhance scientific accuracy, as it would clarify that observed relationships may not imply a causal impact.

 The findings suggest differing influences of variables like FII and COP in the short vs. long term. A deeper exploration of why these factors have opposing impacts over different time horizons could improve interpretative depth.

 3. Expand the Literature Review and Comparison

 Comparing your findings to those of similar studies in emerging markets or other Asian economies can underscore the unique or common effects of macroeconomic variables in the Indian context. Specifically, mention studies on how money supply, exchange rates, and FII impact stock prices in comparable economies, discussing any consistencies or contradictions.

  Many of the policy recommendations align with existing government initiatives. Strengthen this section by drawing connections with specific studies or historical examples where similar macroeconomic interventions (e.g., exchange rate management or increased FII) led to observable outcomes.

 4. Improve the Presentation of Results

  Visual representations (e.g., impulse response functions or time-series graphs of each variable’s impact over time) could improve the clarity of your results, especially for capturing short-run vs. long-run effects.

 Ensure that the significance levels (p-values) of each coefficient in the ARDL model are explicitly reported. This will allow readers to gauge the reliability of the findings on variables with high and low significance.

 5. Recommendations Section

 Greater Specificity

 The recommendation for RBI to control money supply is insightful but could be more actionable. Suggest specific measures that RBI could adopt to balance money supply without triggering inflation, perhaps by referencing open market operations or reserve requirements.

  Expand on your recommendation for export support by discussing concrete steps, such as incentivizing export-oriented sectors or adopting trade policies aligned with ‘Make in India’ and ‘Atmanirbhar Bharat’ initiatives.

 6.  Address Potential Limitation

 Variable Selection and Omitted Variables: Mention any limitations related to variable selection, such as the exclusion of additional external factors like global economic events or investor sentiment, which could influence the stock market. Additionally, a brief statement on potential multicollinearity concerns would reassure readers of the model’s robustness.

 Acknowledge if there are any limitations in the data period that might affect generalizability, especially if there were any structural changes in the economy (e.g., policy changes, COVID-19).

 Essential Revisions for Scientific Soundness

1. Justify the choice of the ARDL model, including brief references to other possible models.
2. Provide precise details on the diagnostic tests and their results.
3. Expand on limitations related to causality and variable selection.
4. Include significance levels for ARDL coefficients in tables for transparency.

Implementing these points will improve the article’s scientific rigour, ensuring that its findings and recommendations are well-supported and its limitations are transparently addressed.

Is the work clearly and accurately presented and does it cite the current literature?

Yes

If applicable, is the statistical analysis and its interpretation appropriate?

Yes

Are all the source data underlying the results available to ensure full reproducibility?

No

Is the study design appropriate and is the work technically sound?

Partly

Are the conclusions drawn adequately supported by the results?

Yes

Are sufficient details of methods and analysis provided to allow replication by others?

Yes

Reviewer Expertise:

Dr. S. Baranidharan specializes in financial analytics, with a focus on stock market analysis, financial econometrics, and economic modeling. His expertise includes hedging strategies, derivative markets, and the application of econometric methods for financial risk management. Dr. Baranidharan is also well-versed in analyzing macroeconomic variables affecting financial markets, particularly within the Indian context. His research contributes to understanding asset pricing, market efficiency, and the practical applications of quantitative finance. With extensive publications, he actively explores innovations in finance education and data-driven decision-making.

I confirm that I have read this submission and believe that I have an appropriate level of expertise to confirm that it is of an acceptable scientific standard, however I have significant reservations, as outlined above.

# Response

**Published:** 2024-11-11

**Authors:** Sanjay Chauhan

Respected Sir,

 With due respect I would like to thank you for your valuable suggestions which I will be incorporating once get access by F1000.

 Thanks and regards

 Sanjay Singh Chauhan
